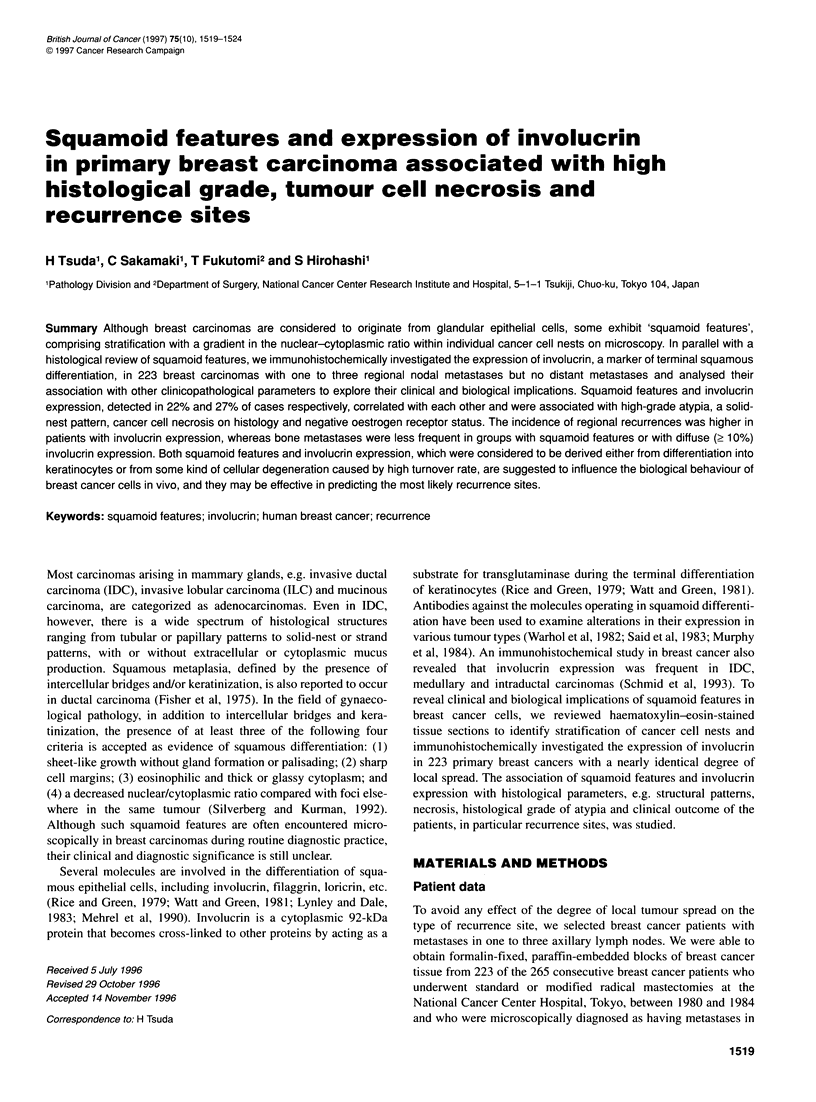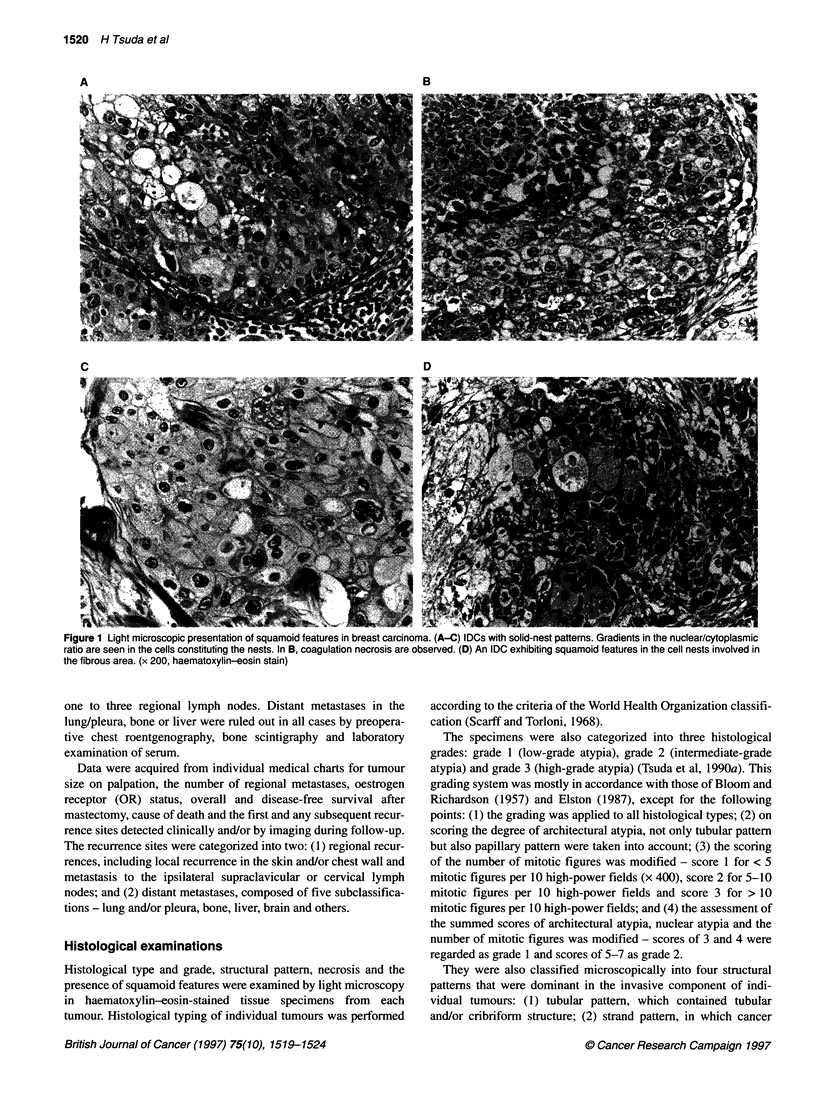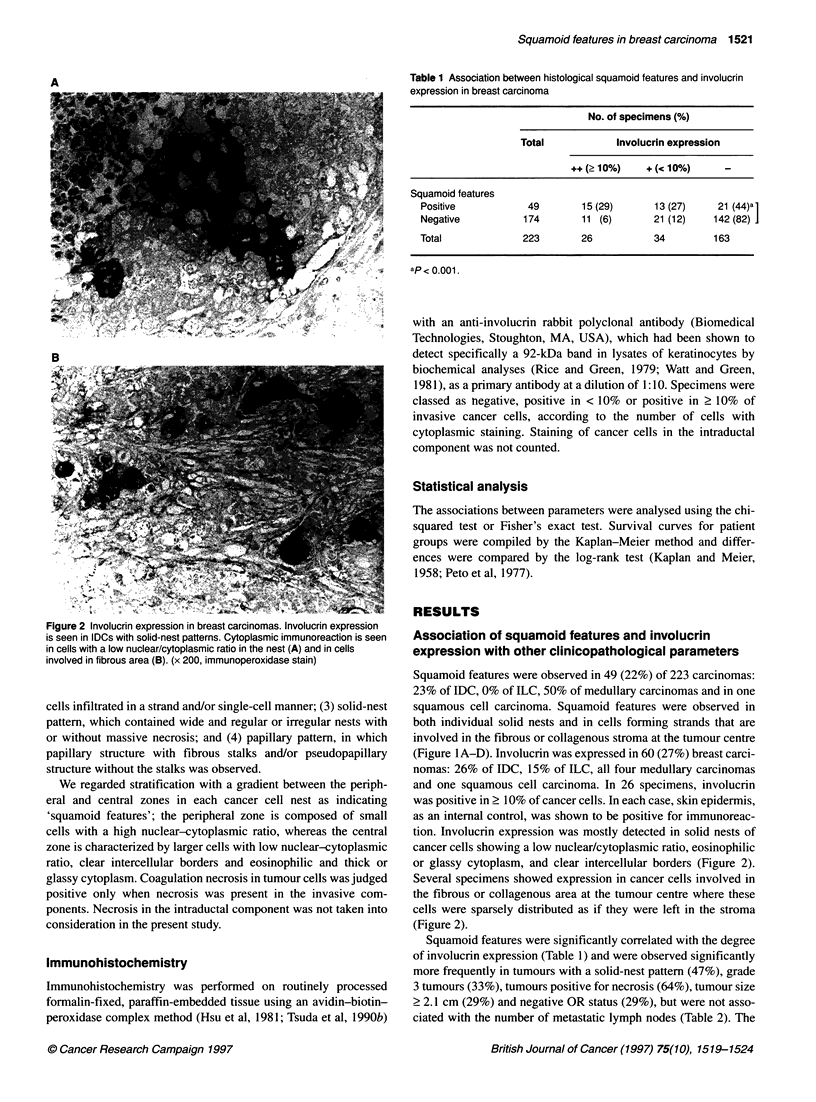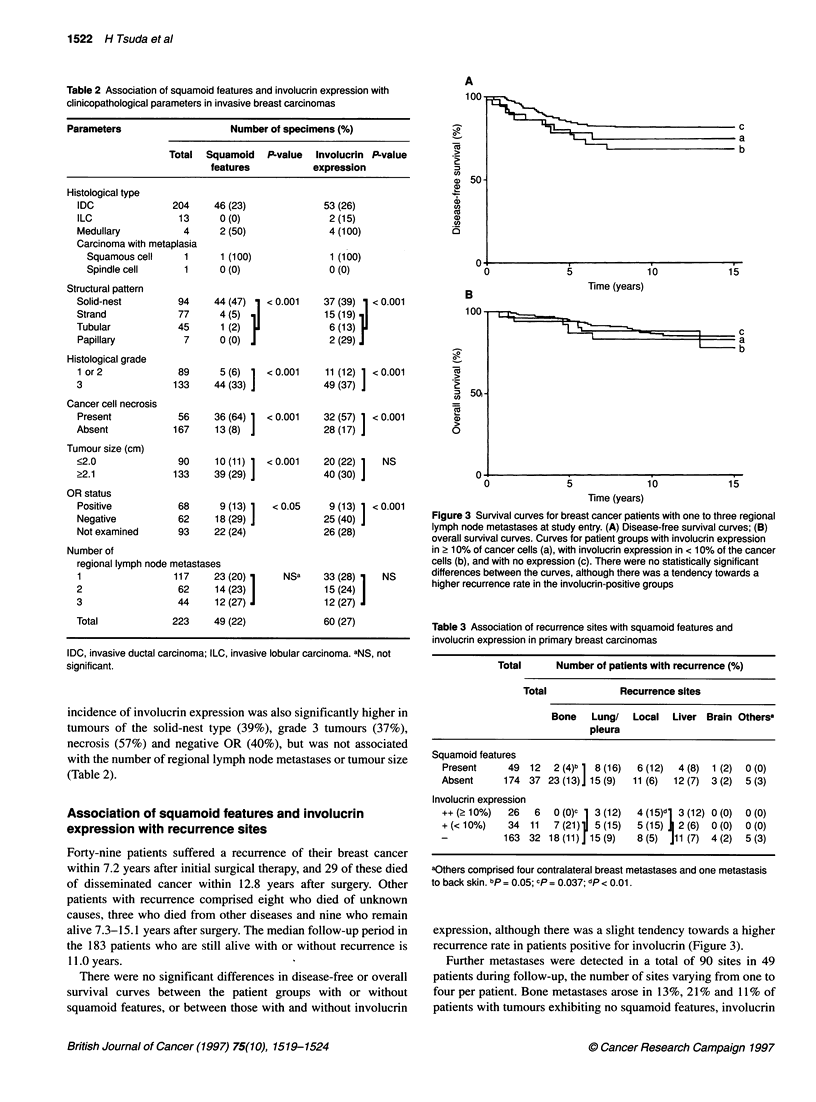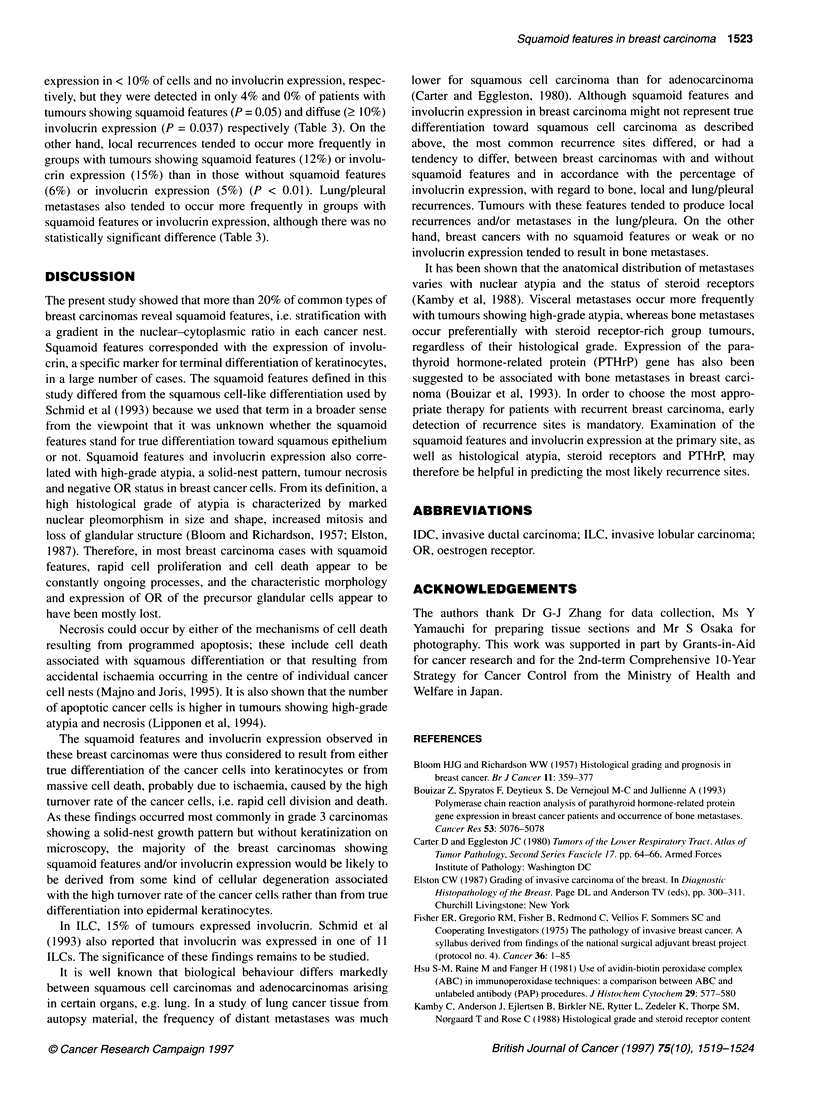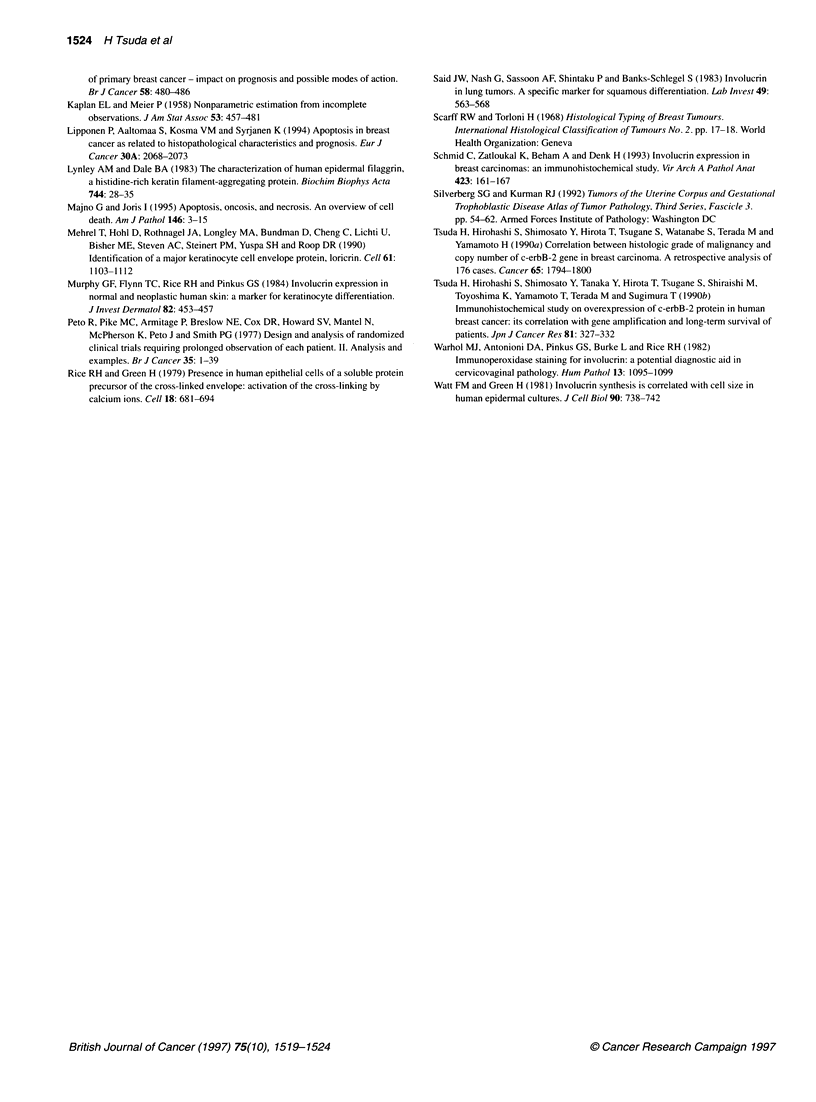# Squamoid features and expression of involucrin in primary breast carcinoma associated with high histological grade, tumour cell necrosis and recurrence sites.

**DOI:** 10.1038/bjc.1997.260

**Published:** 1997

**Authors:** H. Tsuda, C. Sakamaki, T. Fukutomi, S. Hirohashi

**Affiliations:** Pathology Division, National Cancer Center Research Institute and Hospital, Chuo-ku, Tokyo, Japan.

## Abstract

**Images:**


British Journal of Cancer (1997) 75(10), 1519–1524
© 1997 Cancer Research Campaign

### Squamoid features and expression of involucrin in primary breast carcinoma associated with high histological grade, tumour cell necrosis and recurrence sites

**H Tsuda[1], C Sakamaki[1], T Fukutomi[2] and S Hirohashi[1]**

[1]Pathology Division and [2]Department of Surgery, National Cancer Center Research Institute and Hospital, 5–1–1 Tsukiji, Chuo-ku, Tokyo 104, Japan

**Summary** Although breast carcinomas are considered to originate from glandular epithelial cells, some exhibit 'squamoid features', comprising stratification with a gradient in the nuclear–cytoplasmic ratio within individual cancer cell nests on microscopy. In parallel with a histological review of squamoid features, we immunohistochemically investigated the expression of involucrin, a marker of terminal squamous differentiation, in 223 breast carcinomas with one to three regional nodal metastases but no distant metastases and analysed their association with other clinicopathological parameters to explore their clinical and biological implications. Squamoid features and involucrin expression, detected in 22% and 27% of cases respectively, correlated with each other and were associated with high-grade atypia, a solid-nest pattern, cancer cell necrosis on histology and negative oestrogen receptor status. The incidence of regional recurrences was higher in patients with involucrin expression, whereas bone metastases were less frequent in groups with squamoid features or with diffuse (≥ 10%) involucrin expression. Both squamoid features and involucrin expression, which were considered to be derived either from differentiation into keratinocytes or from some kind of cellular degeneration caused by high turnover rate, are suggested to influence the biological behaviour of breast cancer cells in vivo, and they may be effective in predicting the most likely recurrence sites.

**Keywords:** squamoid features; involucrin; human breast cancer; recurrence

Most carcinomas arising in mammary glands, e.g. invasive ductal carcinoma (IDC), invasive lobular carcinoma (ILC) and mucinous carcinoma, are categorized as adenocarcinomas. Even in IDC, however, there is a wide spectrum of histological structures ranging from tubular or papillary patterns to solid-nest or strand patterns, with or without extracellular or cytoplasmic mucus production. Squamous metaplasia, defined by the presence of intercellular bridges and/or keratinization, is also reported to occur in ductal carcinoma (Fisher et al, 1975). In the field of gynaecological pathology, in addition to intercellular bridges and keratinization, the presence of at least three of the following four criteria is accepted as evidence of squamous differentiation: (1) sheet-like growth without gland formation or palisading; (2) sharp cell margins; (3) eosinophilic and thick or glassy cytoplasm; and (4) a decreased nuclear/cytoplasmic ratio compared with foci elsewhere in the same tumour (Silverberg and Kurman, 1992). Although such squamoid features are often encountered microscopically in breast carcinomas during routine diagnostic practice, their clinical and diagnostic significance is still unclear.

Several molecules are involved in the differentiation of squamous epithelial cells, including involucrin, filaggrin, loricrin, etc. (Rice and Green, 1979; Watt and Green, 1981; Lynley and Dale, 1983; Mehrel et al, 1990). Involucrin is a cytoplasmic 92-kDa protein that becomes cross-linked to other proteins by acting as a substrate for transglutaminase during the terminal differentiation of keratinocytes (Rice and Green, 1979; Watt and Green, 1981). Antibodies against the molecules operating in squamoid differentiation have been used to examine alterations in their expression in various tumour types (Warhol et al, 1982; Said et al, 1983; Murphy et al, 1984). An immunohistochemical study in breast cancer also revealed that involucrin expression was frequent in IDC, medullary and intraductal carcinomas (Schmid et al, 1993). To reveal clinical and biological implications of squamoid features in breast cancer cells, we reviewed haematoxylin–eosin-stained tissue sections to identify stratification of cancer cell nests and immunohistochemically investigated the expression of involucrin in 223 primary breast cancers with a nearly identical degree of local spread. The association of squamoid features and involucrin expression with histological parameters, e.g. structural patterns, necrosis, histological grade of atypia and clinical outcome of the patients, in particular recurrence sites, was studied.

### MATERIALS AND METHODS

### Patient data

To avoid any effect of the degree of local tumour spread on the type of recurrence site, we selected breast cancer patients with metastases in one to three axillary lymph nodes. We were able to obtain formalin-fixed, paraffin-embedded blocks of breast cancer tissue from 223 of the 265 consecutive breast cancer patients who underwent standard or modified radical mastectomies at the National Cancer Center Hospital, Tokyo, between 1980 and 1984 and who were microscopically diagnosed as having metastases in

*Received 5 July 1996*
*Revised 29 October 1996*
*Accepted 14 November 1996*

*Correspondence to:* H Tsuda

**Figure 1** Light microscopic presentation of squamoid features in breast carcinoma. (**A–C**) IDCs with solid-nest patterns. Gradients in the nuclear/cytoplasmic ratio are seen in the cells constituting the nests. In **B**, coagulation necrosis are observed. (**D**) An IDC exhibiting squamoid features in the cell nests involved in the fibrous area. (× 200, haematoxylin–eosin stain)

one to three regional lymph nodes. Distant metastases in the lung/pleura, bone or liver were ruled out in all cases by preoperative chest roentgenography, bone scintigraphy and laboratory examination of serum.

Data were acquired from individual medical charts for tumour size on palpation, the number of regional metastases, oestrogen receptor (OR) status, overall and disease-free survival after mastectomy, cause of death and the first and any subsequent recurrence sites detected clinically and/or by imaging during follow-up. The recurrence sites were categorized into two: (1) regional recurrences, including local recurrence in the skin and/or chest wall and metastasis to the ipsilateral supraclavicular or cervical lymph nodes; and (2) distant metastases, composed of five subclassifications – lung and/or pleura, bone, liver, brain and others.

### Histological examinations

Histological type and grade, structural pattern, necrosis and the presence of squamoid features were examined by light microscopy in haematoxylin–eosin-stained tissue specimens from each tumour. Histological typing of individual tumours was performed according to the criteria of the World Health Organization classification (Scarff and Torloni, 1968).

The specimens were also categorized into three histological grades: grade 1 (low-grade atypia), grade 2 (intermediate-grade atypia) and grade 3 (high-grade atypia) (Tsuda et al, 1990*a*). This grading system was mostly in accordance with those of Bloom and Richardson (1957) and Elston (1987), except for the following points: (1) the grading was applied to all histological types; (2) on scoring the degree of architectural atypia, not only tubular pattern but also papillary pattern were taken into account; (3) the scoring of the number of mitotic figures was modified – score 1 for < 5 mitotic figures per 10 high-power fields (× 400), score 2 for 5–10 mitotic figures per 10 high-power fields and score 3 for > 10 mitotic figures per 10 high-power fields; and (4) the assessment of the summed scores of architectural atypia, nuclear atypia and the number of mitotic figures was modified – scores of 3 and 4 were regarded as grade 1 and scores of 5–7 as grade 2.

They were also classified microscopically into four structural patterns that were dominant in the invasive component of individual tumours: (1) tubular pattern, which contained tubular and/or cribriform structure; (2) strand pattern, in which cancer

 © Cancer Research Campaign 1997

Figure 2 Involucrin expression in breast carcinomas. Involucrin expression is seen in IDCs with solid-nest patterns. Cytoplasmic immunoreaction is seen in cells with a low nuclear/cytoplasmic ratio in the nest (A) and in cells involved in fibrous area (B). (× 200, immunoperoxidase stain)

cells infiltrated in a strand and/or single-cell manner; (3) solid-nest pattern, which contained wide and regular or irregular nests with or without massive necrosis; and (4) papillary pattern, in which papillary structure with fibrous stalks and/or pseudopapillary structure without the stalks was observed.

We regarded stratification with a gradient between the peripheral and central zones in each cancer cell nest as indicating 'squamoid features'; the peripheral zone is composed of small cells with a high nuclear–cytoplasmic ratio, whereas the central zone is characterized by larger cells with low nuclear–cytoplasmic ratio, clear intercellular borders and eosinophilic and thick or glassy cytoplasm. Coagulation necrosis in tumour cells was judged positive only when necrosis was present in the invasive components. Necrosis in the intraductal component was not taken into consideration in the present study.

### Immunohistochemistry

Immunohistochemistry was performed on routinely processed formalin-fixed, paraffin-embedded tissue using an avidin–biotin–peroxidase complex method (Hsu et al, 1981; Tsuda et al, 1990*b*) with an anti-involucrin rabbit polyclonal antibody (Biomedical Technologies, Stoughton, MA, USA), which had been shown to detect specifically a 92-kDa band in lysates of keratinocytes by biochemical analyses (Rice and Green, 1979; Watt and Green, 1981), as a primary antibody at a dilution of 1:10. Specimens were classed as negative, positive in < 10% or positive in ≥ 10% of invasive cancer cells, according to the number of cells with cytoplasmic staining. Staining of cancer cells in the intraductal component was not counted.

**Table 1** Association between histological squamoid features and involucrin expression in breast carcinoma

| | No. of specimens (%) | | | |
|---|---|---|---|---|
| | Total | Involucrin expression | | |
| | | ++ (≥ 10%) | + (< 10%) | – |
| Squamoid features | | | | |
| Positive | 49 | 15 (29) | 13 (27) | 21 (44)[a] |
| Negative | 174 | 11 (6) | 21 (12) | 142 (82) |
| Total | 223 | 26 | 34 | 163 |

[a]$P < 0.001$.

### Statistical analysis

The associations between parameters were analysed using the chi-squared test or Fisher's exact test. Survival curves for patient groups were compiled by the Kaplan–Meier method and differences were compared by the log-rank test (Kaplan and Meier, 1958; Peto et al, 1977).

### RESULTS

### Association of squamoid features and involucrin expression with other clinicopathological parameters

Squamoid features were observed in 49 (22%) of 223 carcinomas: 23% of IDC, 0% of ILC, 50% of medullary carcinomas and in one squamous cell carcinoma. Squamoid features were observed in both individual solid nests and in cells forming strands that are involved in the fibrous or collagenous stroma at the tumour centre (Figure 1A–D). Involucrin was expressed in 60 (27%) breast carcinomas: 26% of IDC, 15% of ILC, all four medullary carcinomas and one squamous cell carcinoma. In 26 specimens, involucrin was positive in ≥ 10% of cancer cells. In each case, skin epidermis, as an internal control, was shown to be positive for immunoreaction. Involucrin expression was mostly detected in solid nests of cancer cells showing a low nuclear/cytoplasmic ratio, eosinophilic or glassy cytoplasm, and clear intercellular borders (Figure 2). Several specimens showed expression in cancer cells involved in the fibrous or collagenous area at the tumour centre where these cells were sparsely distributed as if they were left in the stroma (Figure 2).

Squamoid features were significantly correlated with the degree of involucrin expression (Table 1) and were observed significantly more frequently in tumours with a solid-nest pattern (47%), grade 3 tumours (33%), tumours positive for necrosis (64%), tumour size ≥ 2.1 cm (29%) and negative OR status (29%), but were not associated with the number of metastatic lymph nodes (Table 2). The

© Cancer Research Campaign 1997

Table 2 Association of squamoid features and involucrin expression with clinicopathological parameters in invasive breast carcinomas

| Parameters | Total | Squamoid features | P-value | Involucrin expression | P-value |
|---|---|---|---|---|---|
| | | Number of specimens (%) | | | |
| Histological type | | | | | |
| IDC | 204 | 46 (23) | | 53 (26) | |
| ILC | 13 | 0 (0) | | 2 (15) | |
| Medullary | 4 | 2 (50) | | 4 (100) | |
| Carcinoma with metaplasia | | | | | |
| Squamous cell | 1 | 1 (100) | | 1 (100) | |
| Spindle cell | 1 | 0 (0) | | 0 (0) | |
| Structural pattern | | | | | |
| Solid-nest | 94 | 44 (47) | < 0.001 | 37 (39) | < 0.001 |
| Strand | 77 | 4 (5) | | 15 (19) | |
| Tubular | 45 | 1 (2) | | 6 (13) | |
| Papillary | 7 | 0 (0) | | 2 (29) | |
| Histological grade | | | | | |
| 1 or 2 | 89 | 5 (6) | < 0.001 | 11 (12) | < 0.001 |
| 3 | 133 | 44 (33) | | 49 (37) | |
| Cancer cell necrosis | | | | | |
| Present | 56 | 36 (64) | < 0.001 | 32 (57) | < 0.001 |
| Absent | 167 | 13 (8) | | 28 (17) | |
| Tumour size (cm) | | | | | |
| ≤2.0 | 90 | 10 (11) | < 0.001 | 20 (22) | NS |
| ≥2.1 | 133 | 39 (29) | | 40 (30) | |
| OR status | | | | | |
| Positive | 68 | 9 (13) | < 0.05 | 9 (13) | < 0.001 |
| Negative | 62 | 18 (29) | | 25 (40) | |
| Not examined | 93 | 22 (24) | | 26 (28) | |
| Number of regional lymph node metastases | | | | | |
| 1 | 117 | 23 (20) | NS[a] | 33 (28) | NS |
| 2 | 62 | 14 (23) | | 15 (24) | |
| 3 | 44 | 12 (27) | | 12 (27) | |
| Total | 223 | 49 (22) | | 60 (27) | |

IDC, invasive ductal carcinoma; ILC, invasive lobular carcinoma. [a]NS, not significant.

incidence of involucrin expression was also significantly higher in tumours of the solid-nest type (39%), grade 3 tumours (37%), necrosis (57%) and negative OR (40%), but was not associated with the number of regional lymph node metastases or tumour size (Table 2).

### Association of squamoid features and involucrin expression with recurrence sites

Forty-nine patients suffered a recurrence of their breast cancer within 7.2 years after initial surgical therapy, and 29 of these died of disseminated cancer within 12.8 years after surgery. Other patients with recurrence comprised eight who died of unknown causes, three who died from other diseases and nine who remain alive 7.3–15.1 years after surgery. The median follow-up period in the 183 patients who are still alive with or without recurrence is 11.0 years.

There were no significant differences in disease-free or overall survival curves between the patient groups with or without squamoid features, or between those with and without involucrin expression, although there was a slight tendency towards a higher recurrence rate in patients positive for involucrin (Figure 3).

Further metastases were detected in a total of 90 sites in 49 patients during follow-up, the number of sites varying from one to four per patient. Bone metastases arose in 13%, 21% and 11% of patients with tumours exhibiting no squamoid features, involucrin

Figure 3 Survival curves for breast cancer patients with one to three regional lymph node metastases at study entry. (A) Disease-free survival curves; (B) overall survival curves. Curves for patient groups with involucrin expression in ≥ 10% of cancer cells (a), with involucrin expression in < 10% of the cancer cells (b), and with no expression (c). There were no statistically significant differences between the curves, although there was a tendency towards a higher recurrence rate in the involucrin-positive groups

Table 3 Association of recurrence sites with squamoid features and involucrin expression in primary breast carcinomas

| | Total | Number of patients with recurrence (%) | | | | | | |
|---|---|---|---|---|---|---|---|---|
| | | Total | Recurrence sites | | | | | |
| | | | Bone | Lung/pleura | Local | Liver | Brain | Others[a] |
| Squamoid features | | | | | | | | |
| Present | 49 | 12 | 2 (4)[b] | 8 (16) | 6 (12) | 4 (8) | 1 (2) | 0 (0) |
| Absent | 174 | 37 | 23 (13) | 15 (9) | 11 (6) | 12 (7) | 3 (2) | 5 (3) |
| Involucrin expression | | | | | | | | |
| ++ (≥ 10%) | 26 | 6 | 0 (0)[c] | 3 (12) | 4 (15)[d] | 3 (12) | 0 (0) | 0 (0) |
| + (< 10%) | 34 | 11 | 7 (21) | 5 (15) | 5 (15) | 2 (6) | 0 (0) | 0 (0) |
| – | 163 | 32 | 18 (11) | 15 (9) | 8 (5) | 11 (7) | 4 (2) | 5 (3) |

[a]Others comprised four contralateral breast metastases and one metastasis to back skin. [b]$P = 0.05$; [c]$P = 0.037$; [d]$P < 0.01$.

© Cancer Research Campaign 1997

expression in < 10% of cells and no involucrin expression, respectively, but they were detected in only 4% and 0% of patients with tumours showing squamoid features ($P = 0.05$) and diffuse (≥ 10%) involucrin expression ($P = 0.037$) respectively (Table 3). On the other hand, local recurrences tended to occur more frequently in groups with tumours showing squamoid features (12%) or involucrin expression (15%) than in those without squamoid features (6%) or involucrin expression (5%) ($P < 0.01$). Lung/pleural metastases also tended to occur more frequently in groups with squamoid features or involucrin expression, although there was no statistically significant difference (Table 3).

### DISCUSSION

The present study showed that more than 20% of common types of breast carcinomas reveal squamoid features, i.e. stratification with a gradient in the nuclear–cytoplasmic ratio in each cancer nest. Squamoid features corresponded with the expression of involucrin, a specific marker for terminal differentiation of keratinocytes, in a large number of cases. The squamoid features defined in this study differed from the squamous cell-like differentiation used by Schmid et al (1993) because we used that term in a broader sense from the viewpoint that it was unknown whether the squamoid features stand for true differentiation toward squamous epithelium or not. Squamoid features and involucrin expression also correlated with high-grade atypia, a solid-nest pattern, tumour necrosis and negative OR status in breast cancer cells. From its definition, a high histological grade of atypia is characterized by marked nuclear pleomorphism in size and shape, increased mitosis and loss of glandular structure (Bloom and Richardson, 1957; Elston, 1987). Therefore, in most breast carcinoma cases with squamoid features, rapid cell proliferation and cell death appear to be constantly ongoing processes, and the characteristic morphology and expression of OR of the precursor glandular cells appear to have been mostly lost.

Necrosis could occur by either of the mechanisms of cell death resulting from programmed apoptosis; these include cell death associated with squamous differentiation or that resulting from accidental ischaemia occurring in the centre of individual cancer cell nests (Majno and Joris, 1995). It is also shown that the number of apoptotic cancer cells is higher in tumours showing high-grade atypia and necrosis (Lipponen et al, 1994).

The squamoid features and involucrin expression observed in these breast carcinomas were thus considered to result from either true differentiation of the cancer cells into keratinocytes or from massive cell death, probably due to ischaemia, caused by the high turnover rate of the cancer cells, i.e. rapid cell division and death. As these findings occurred most commonly in grade 3 carcinomas showing a solid-nest growth pattern but without keratinization on microscopy, the majority of the breast carcinomas showing squamoid features and/or involucrin expression would be likely to be derived from some kind of cellular degeneration associated with the high turnover rate of the cancer cells rather than from true differentiation into epidermal keratinocytes.

In ILC, 15% of tumours expressed involucrin. Schmid et al (1993) also reported that involucrin was expressed in one of 11 ILCs. The significance of these findings remains to be studied.

It is well known that biological behaviour differs markedly between squamous cell carcinomas and adenocarcinomas arising in certain organs, e.g. lung. In a study of lung cancer tissue from autopsy material, the frequency of distant metastases was much lower for squamous cell carcinoma than for adenocarcinoma (Carter and Eggleston, 1980). Although squamoid features and involucrin expression in breast carcinoma might not represent true differentiation toward squamous cell carcinoma as described above, the most common recurrence sites differed, or had a tendency to differ, between breast carcinomas with and without squamoid features and in accordance with the percentage of involucrin expression, with regard to bone, local and lung/pleural recurrences. Tumours with these features tended to produce local recurrences and/or metastases in the lung/pleura. On the other hand, breast cancers with no squamoid features or weak or no involucrin expression tended to result in bone metastases.

It has been shown that the anatomical distribution of metastases varies with nuclear atypia and the status of steroid receptors (Kamby et al, 1988). Visceral metastases occur more frequently with tumours showing high-grade atypia, whereas bone metastases occur preferentially with steroid receptor-rich group tumours, regardless of their histological grade. Expression of the parathyroid hormone-related protein (PTHrP) gene has also been suggested to be associated with bone metastases in breast carcinoma (Bouizar et al, 1993). In order to choose the most appropriate therapy for patients with recurrent breast carcinoma, early detection of recurrence sites is mandatory. Examination of the squamoid features and involucrin expression at the primary site, as well as histological atypia, steroid receptors and PTHrP, may therefore be helpful in predicting the most likely recurrence sites.

### ABBREVIATIONS

IDC, invasive ductal carcinoma; ILC, invasive lobular carcinoma; OR, oestrogen receptor.

### ACKNOWLEDGEMENTS

The authors thank Dr G-J Zhang for data collection, Ms Y Yamauchi for preparing tissue sections and Mr S Osaka for photography. This work was supported in part by Grants-in-Aid for cancer research and for the 2nd-term Comprehensive 10-Year Strategy for Cancer Control from the Ministry of Health and Welfare in Japan.

### REFERENCES

Bloom HJG and Richardson WW (1957) Histological grading and prognosis in breast cancer. *Br J Cancer* **11**: 359–377

Bouizar Z, Spyratos F, Deytieux S, De Vernejoul M-C and Jullienne A (1993) Polymerase chain reaction analysis of parathyroid hormone-related protein gene expression in breast cancer patients and occurrence of bone metastases. *Cancer Res* **53**: 5076–5078

Carter D and Eggleston JC (1980) *Tumors of the Lower Respiratory Tract. Atlas of Tumor Pathology, Second Series Fascicle 17.* pp. 64–66. Armed Forces Institute of Pathology: Washington DC

Elston CW (1987) Grading of invasive carcinoma of the breast. In *Diagnostic Histopathology of the Breast.* Page DL and Anderson TV (eds), pp. 300–311. Churchill Livingstone: New York

Fisher ER, Gregorio RM, Fisher B, Redmond C, Vellios F, Sommers SC and Cooperating Investigators (1975) The pathology of invasive breast cancer. A syllabus derived from findings of the national surgical adjuvant breast project (protocol no. 4). *Cancer* **36**: 1–85

Hsu S-M, Raine M and Fanger H (1981) Use of avidin-biotin peroxidase complex (ABC) in immunoperoxidase techniques: a comparison between ABC and unlabeled antibody (PAP) procedures. *J Histochem Cytochem* **29**: 577–580

Kamby C, Anderson J, Ejlertsen B, Birkler NE, Rytter L, Zedeler K, Thorpe SM, Nørgaard T and Rose C (1988) Histological grade and steroid receptor content

© Cancer Research Campaign 1997

of primary breast cancer – impact on prognosis and possible modes of action. *Br J Cancer* **58**: 480–486

Kaplan EL and Meier P (1958) Nonparametric estimation from incomplete observations. *J Am Stat Assoc* **53**: 457–481

Lipponen P, Aaltomaa S, Kosma VM and Syrjanen K (1994) Apoptosis in breast cancer as related to histopathological characteristics and prognosis. *Eur J Cancer* **30A**: 2068–2073

Lynley AM and Dale BA (1983) The characterization of human epidermal filaggrin, a histidine-rich keratin filament-aggregating protein. *Biochim Biophys Acta* **744**: 28–35

Majno G and Joris I (1995) Apoptosis, oncosis, and necrosis. An overview of cell death. *Am J Pathol* **146**: 3–15

Mehrel T, Hohl D, Rothnagel JA, Longley MA, Bundman D, Cheng C, Lichti U, Bisher ME, Steven AC, Steinert PM, Yuspa SH and Roop DR (1990) Identification of a major keratinocyte cell envelope protein, loricrin. *Cell* **61**: 1103–1112

Murphy GF, Flynn TC, Rice RH and Pinkus GS (1984) Involucrin expression in normal and neoplastic human skin: a marker for keratinocyte differentiation. *J Invest Dermatol* **82**: 453–457

Peto R, Pike MC, Armitage P, Breslow NE, Cox DR, Howard SV, Mantel N, McPherson K, Peto J and Smith PG (1977) Design and analysis of randomized clinical trials requiring prolonged observation of each patient. II. Analysis and examples. *Br J Cancer* **35**: 1–39

Rice RH and Green H (1979) Presence in human epithelial cells of a soluble protein precursor of the cross-linked envelope: activation of the cross-linking by calcium ions. *Cell* **18**: 681–694

Said JW, Nash G, Sassoon AF, Shintaku P and Banks-Schlegel S (1983) Involucrin in lung tumors. A specific marker for squamous differentiation. *Lab Invest* **49**: 563–568

Scarff RW and Torloni H (1968) *Histological Typing of Breast Tumours. International Histological Classification of Tumours No. 2*. pp. 17–18. World Health Organization: Geneva

Schmid C, Zatloukal K, Beham A and Denk H (1993) Involucrin expression in breast carcinomas: an immunohistochemical study. *Vir Arch A Pathol Anat* **423**: 161–167

Silverberg SG and Kurman RJ (1992) *Tumors of the Uterine Corpus and Gestational Trophoblastic Disease Atlas of Tumor Pathology, Third Series, Fascicle 3*. pp. 54–62. Armed Forces Institute of Pathology: Washington DC

Tsuda H, Hirohashi S, Shimosato Y, Hirota T, Tsugane S, Watanabe S, Terada M and Yamamoto H (1990*a*) Correlation between histologic grade of malignancy and copy number of c-erbB-2 gene in breast carcinoma. A retrospective analysis of 176 cases. *Cancer* **65**: 1794–1800

Tsuda H, Hirohashi S, Shimosato Y, Tanaka Y, Hirota T, Tsugane S, Shiraishi M, Toyoshima K, Yamamoto T, Terada M and Sugimura T (1990*b*) Immunohistochemical study on overexpression of c-erbB-2 protein in human breast cancer: its correlation with gene amplification and long-term survival of patients. *Jpn J Cancer Res* **81**: 327–332

Warhol MJ, Antonioni DA, Pinkus GS, Burke L and Rice RH (1982) Immunoperoxidase staining for involucrin: a potential diagnostic aid in cervicovaginal pathology. *Hum Pathol* **13**: 1095–1099

Watt FM and Green H (1981) Involucrin synthesis is correlated with cell size in human epidermal cultures. *J Cell Biol* **90**: 738–742

*© Cancer Research Campaign 1997*